# Supplementary material for: Using the nose as a factory to secrete proteins into the lungs or circulation
Source: Mol Ther Adv. 2026 Apr 6;34(2):201733. doi: 10.1016/j.omta.2026.201733 (PMC13144561; doi:10.1016/j.omta.2026.201733)
Supplement: Document S1. Figures S1 and S2 and Tables S1 and S2 [file mmc1.pdf]

OMTA, Volume *34*

Supplemental information

Using the nose as a factory to secrete proteins into the lungs or circulation

Anthony Sinadinos, Robyn Bell, Claudia Ivette Juarez-Molina, Cuixiang Meng, Emily Castells, Mariana A. Viegas, Deborah R. Gill, Stephen C. Hyde, Uta Griesenbach, and Eric W.F.W. Alton

Table S1. Cellular level pathology parsed from haematoxylin and eosin stained transverse nasal-tissue sections from multiple small volume lentiviral vector (F.H/N), saline (TSSM), and untreated (sham) individualised mice, scored at 1 day post dosing. Nasal tissue pathology summarised for each mouse spanning five transverse levels of nose. Key: 0 normal and clear; + very limited and focal immune infiltrate; ++ more pronounced but focal infiltrate; +++ multifocal and/or infrequent prominent infiltrate. '/' indicated borderline representation. x focal blebbing with occasional loss of cells and/or infrequent keratinisation; xx more pronounced but sporadic blebbing and cell loss and/or pronounced keratinisation; xxx multifocal and pronounced blebbing and cell loss. Grades: 0 = within normal limits; 1 = mild focal structural changes with some to no inflammation; 2 = moderate range of mucosal changes with mild focused inflammation.

Nasal tissue									
		Sample	Frontal Nares (structure)	Lower level mucosa (structure)	Sub-mucosal blood vessel dilation	Macrophages	Neutrophils	Lymphocytes	Grade
1 day	Anaesthesia only	sham_1	0	0	-	0	0	0	0
		sham_2	0	xx	-	0	++	0	1-2
		sham_3	0	0	-	0	++	0	2
		sham_4	0	xx	-	0	++	0	2
		sham_5	0	0	-	0	++	0	1-2
	10 x 5µL over 50mins	F.H/N_1	x (keratinisation)	0	yes	0	++	++	2
		F.H/N_2	0	0	-	0	++	++	2
		F.H/N_3	0	0	yes	0	0	0	0-1
		F.H/N_4	0	xx	yes	0	0	0	1-2
		F.H/N_5	0	0	-	0	0	0	0
		TSSM_1	0	0	-	+	++	++	1
		TSSM_2	0	0	yes	0	++	0	2
		TSSM_3	0	xx	-	0	++	++	2
		TSSM_4	0	0	-	0	+	0	1
		TSSM_5	0	0	-	0	0	0	0

Table S2. Cellular level pathology parsed from haematoxylin and eosin stained lung transverse tissue sections from multiple small volume lentiviral vector (F.H/N), saline (TSSM), and untreated (sham) individualised mice, scored at 1 and 7 days post dosing. Lung tissue pathology summarised for each mouse spanning six transverse levels. Key: 0 normal and clear; + very limited and focal immune infiltrate; ++ more pronounced but focal infiltrate; +++ multifocal and/or infrequent prominent infiltrate. '/' indicated borderline representation. x mild and infrequent wall or extracellular space thickening; xx more pronounced but sporadic thickening. Grades: 0 = within normal limits; 1 = limited mild severity/top end of normal changes; 2 = limited severity.

Lungs										
		Sample	Trachea	Lung macrophages	Lung neutrophils	Lung lymphocytes	Eosinophilia	Alveoli	Bronchial lymph nodes	Grade
1 day	Anaesthesia only	sham_1	0	0	0	+	0	0	0	0-1
1 day	Anaesthesia only	sham _2	0	+	0	+	0	0	0	0-1
1 day	Anaesthesia only	sham_3	0	0	0	0	0	0	0	0
1 day	10 x 5µL over 50mins	F.H/N_1	0	0	0	+	0	0	0	1
1 day	10 x 5µL over 50mins	F.H/N_2	0	++	0	+	+	x	0	1-2
1 day	10 x 5µL over 50mins	F.H/N_3	0	+	0	+	0	0	0	1
1 day	10 x 5µL over 50mins	F.H/N_4	0	+	0	0	0	0	0	1
1 day	10 x 5µL over 50mins	F.H/N_5	0	+	0	0	0	x	0	1
1 day	10 x 5µL over 50mins	TSSM_1	0	0	0	+	0	0	0	0-1
1 day	10 x 5µL over 50mins	TSSM_2	0	0	0	+	0	0	0	0
1 day	10 x 5µL over 50mins	TSSM_3	0	0	0	+	0	0	+ (w/ vacuolation)	0
1 day	10 x 5µL over 50mins	TSSM_4	0	0	0	+	0	0	0	0
1 day	10 x 5µL over 50mins	TSSM_5	0	0/+	0	+	0	0	0	0-1
7 days	Anaesthesia only	sham_1	0	0	0	0	0	0	Not scored	0
7 days	Anaesthesia only	sham_2	+	0	0	+	0	0	0	0-1
7 days	Anaesthesia only	sham_3	0	0	0	0	0	0	+	0
7 days	Anaesthesia only	sham_4	0	0	0	0	0	0	+	0
7 days	Anaesthesia only	sham_5	0	0	0	++	0	0	Not scored	1
7 days	10 x 5µL over 50mins	F.H/N_1	0	0	0	+	0	0	++	0-1
7 days	10 x 5µL over 50mins	F.H/N_2	0	0	0	++	0	0	Not scored	1
7 days	10 x 5µL over 50mins	F.H/N_3	0	0	0	0	0	0	Not scored	0

		F.H/N_4	0	0	0	0	0	0	+	0
		F.H/N_5	0	0	0	+	0	0	+	1

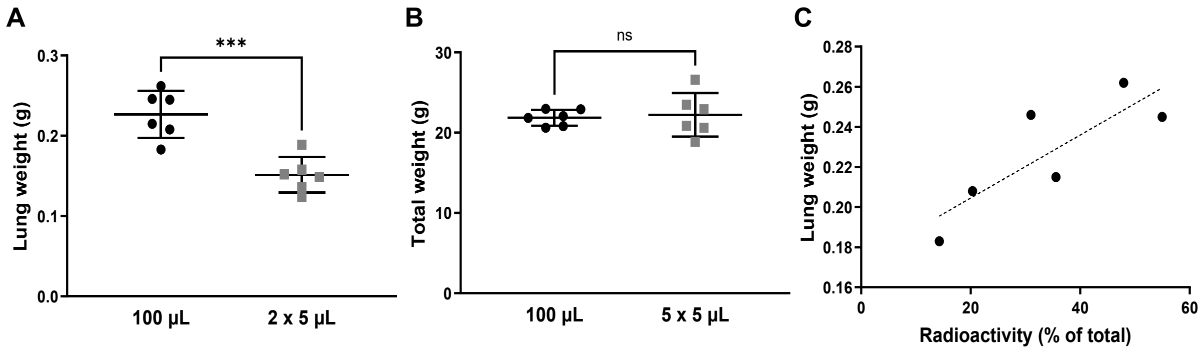

Figure S1. Small volume intranasal dosing of ^{99m}Tc-DTPA restricts fluid distribution to the site of instillation. Female 8-12 week-old mice received either a single 100µL, or two 5µL administrations (separated by 5 minutes) of volume(s) of PBS containing 5MBq of ^{99m}Tc-DTPA by intranasal bolus 'sniffing', and the separate body-areas, head, lungs, and the remaining body, were subsequently measured for radiotracer retention by gamma scintillation. Following dosing and culling, excised (A) lung (t-test, n=6, $p<0.001$) and (B) remaining total (head and body) (t-test, n=6, p=ns) weights were compared (individual values shown along with mean ± SD). (C) For tissue from the 100µL-dosed mice, lung weights were correlated with radioactivity (Pearson, r=0.832, r^2=0.6923, p=0.04). *** = $p<0.001$; ns = non-significant.

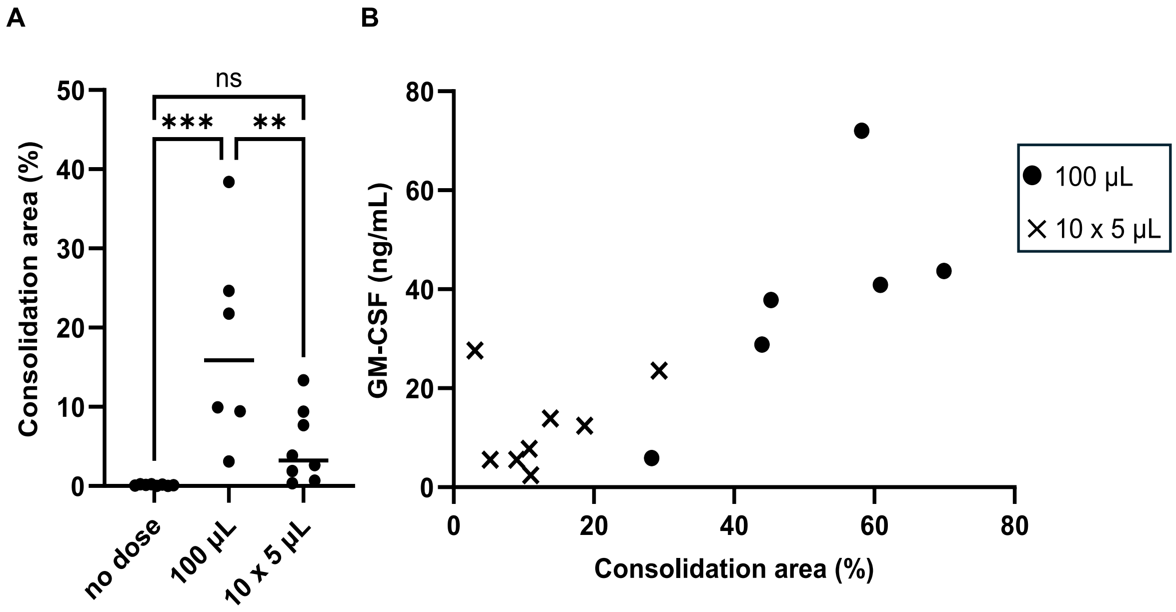

Figure S2. Granulocyte macrophage colony stimulating factor (GM-CSF) related lung consolidation, in Pulmonary Alveolar Proteinosis (PAP) mice, receiving 2.3e8 TU of rSIV.F/HN, encoding a hCEF-driven mouse codon-optimised version of GM-CSF, in either 100 µL or multiple small volume (10 x 5 µL) intranasal applications, measured 2.5 weeks post-dosing. (A) Whole tissue cross-sections from right lung lobes were analysed for consolidated areas of lung (ANOVA, n=8,6,8, F=12.2, DFn=2, DFd=19, p=0.0008: Tukey's post-hoc) (individual values shown along with mean). (B) Left and right lung average consolidation area was compared with bronchoalveolar lavage fluid GM-CSF levels measured from the same lentiviral vector treated mice (Pearson, n=14, r=0.8048, p=0.0005). ** = p<0.005, *** = p<0.0005, ns = non-significant.
