# Supplementary material for: Using the nose as a factory to secrete proteins into the lungs or circulation
Source: Mol Ther Adv. 2026 Apr 6;34(2):201733. doi: 10.1016/j.omta.2026.201733 (PMC13144561; doi:10.1016/j.omta.2026.201733)
Supplement: Document S2. Article plus supplemental information [file mmc2.pdf]

Molecular Therapy
Advances
Original Article

Using the nose as a factory to secrete proteins into the lungs or circulation

Anthony Sinadinos,[1,2] Robyn Bell,[1,2] Claudia Ivette Juarez-Molina,[1,2] Cuixiang Meng,[1,2] Emily Castells,[3,2] Mariana A. Viegas,[3,2] Deborah R. Gill,[3,2] Stephen C. Hyde,[3,2] Uta Griesenbach,[1,2,4] and Eric W.F.W. Alton[1,2,4]

[1]National Heart and Lung Institute, Imperial College London, London SW3 6LY, UK; [2]UK Respiratory Gene Therapy Consortium, London, UK; [3]Radcliffe Department of Medicine, University of Oxford, Oxford OX3 9DS, UK

Targeting of the nasal epithelium for sustained therapeutic protein secretion represents a potential non-invasive lentiviral vector application strategy. Using reporter imaging, molecular, and radiopharmaceutical tracing methods in mice, we have developed an intranasal (nose-only) dosing strategy with a Sendai virus envelope glycoprotein pseudotyped lentiviral vector (rSIV.F/HN). Using multiple (up to 10) small-volume (5 µL) intranasal bolus applications, a technetium radiotracer showed >90% liquid retention in the murine head and <1% in the lung. Following vector administration, transgene expression was dose-related in the nose, with minimal lung expression. No acute nasal toxicity was associated with nose-only delivery. Next, we compared levels of a secreted protein, *Gaussia* luciferase (Gluc), in the airways and serum after nose-only and intravenous administration of rSIV.F/HN-Gluc (2e8 TU/mouse). Gluc expression in the nose and lungs was higher following nose-only versus intravenous administration. Serum levels were similar after either route of administration. Finally, nose-only delivery of rSIV.F/HN encoding granulocyte-macrophage colony-stimulating factor (GM-CSF) led to sufficient lung levels of this therapeutic protein to correct disease biomarkers in a mouse model of pulmonary alveolar proteinosis. We conclude that non-invasive administration of a lentiviral vector to the nasal epithelium provides a safe and convenient route for secreted protein production and is readily translatable into humans.

INTRODUCTION

Transgenic secreted proteins are released from viral vector-transduced cells and may have their effect either locally, close to the site of vector delivery, or after a more widespread distribution. For example, alpha-1 antitrypsin deficiency is a disease that may be treated via the local production of a secreted protein in the lungs,[1] whereas hemophilia may be corrected following factor VIII or IX secretion into the systemic circulatory system.[2,3] In either case, gene therapy-mediated therapeutic protein secretion may provide titratable and long-lasting levels of a secreted protein at the desired site(s) of action.

To date, research has mostly focused on intrapulmonary delivery of gene therapy agents for the treatment of respiratory disorders, but the systemic availability of lung-derived proteins has also been described.[4–6] The lungs represent a protein factory target site with a large surface area and access to the circulation. They are also accessible via minimally invasive delivery, compared with many other organs and tissues. These considerations also apply to the nasal epithelium,[7] raising the possibility of exclusively targeting this region with gene therapy vectors to induce production of a secreted therapeutic protein for subsequent biodistribution to the lungs or the systemic circulation.[8,9]

To explore this approach, we have established an *in vivo* method that preferentially delivers a lentiviral vector to the murine nose. We have previously developed a simian immunodeficiency virus (SIV) vector pseudotyped with Sendai virus F and HN envelope proteins to generate a lentiviral vector (rSIV.F/HN) that allows for the efficient *in vivo* transduction of mammalian pulmonary epithelium and which is now in a first-in-human clinical trial for the treatment of cystic fibrosis.[10–12] Using this vector, we provide evidence for using the nose-only route as a factory to deliver proteins to the lungs. As an exemplar, lentiviral-mediated delivery of granulocyte-macrophage colony-stimulating factor (GM-CSF) produced significant therapeutic benefit in a murine model of autoimmune pulmonary alveolar proteinosis (aPAP). Second, we demonstrate secretion of levels of protein into the systemic circulation similar to those produced following intravenous delivery, providing a likely safer and more facile human administration route.

Received 17 November 2025; accepted 3 April 2026;
https://doi.org/10.1016/j.omta.2026.201733.

[4]Senior author

Correspondence: Uta Griesenbach, National Heart and Lung Institute, Imperial College London, London SW3 6LY, UK.
E-mail: u.griesenbach@imperial.ac.uk

© 2026 The Authors.
Published by Elsevier Inc. on behalf of The American Society of Gene and Cell Therapy.
This is an open access article under the CC BY license (http://creativecommons.org/licenses/by/4.0/).

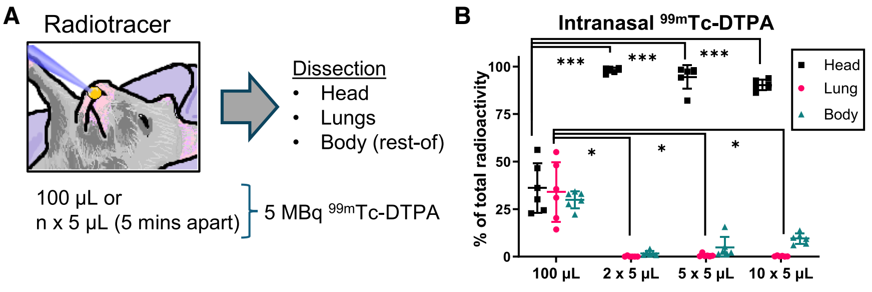

Figure 1. Intranasal dosing of female 8–12 week-old mice with multiple small volumes of saline containing a radioactive tracer

(A) Schematic representation of the study design, in which mice received either a single 100 µL or multiple 5 min-separated 5 µL boluses of a technetium-based radiotracer (^{99m}TC-DTPA), totaling 5 MBq per mouse, before measuring radioactive liquid distribution in partitioned heads, lungs, and remaining body sections. (B) Radioactivity was measured 24–28 h after dosing and tissue collection, comparing body sections from 100 µL treated mice to the same sections in multiple small-volume cohorts (head comparison: Welch's ANOVA, $n = 6$, $p < 0.0001$: Dunnet's post-hoc test; lung comparison: Welch's ANOVA, $n = 6$, $p < 0.005$: Dunnet's post-hoc test). Values are shown as a percentage of the combined body section radioactivity for each mouse (individual values shown along with mean ± SD). * = $p < 0.05$; *** = $p < 0.0005$.

RESULTS

Intranasal dosing of multiple small liquid volumes restricts deposition to the nose

The deposition of an intranasally administered saline solution was tracked via a radiotracer study to assess the relationship between instillation volume and biodistribution through the airway tract of mice. A single 100 µL bolus of saline, containing a ^{99m}Technetium(Tc)-diethylenetriamine pentaacetate (DTPA) tracer (5 MBq), was intranasally administered to mice. This dosing volume represents a typical maximum intranasal application, as previously used in multiple murine studies to deliver vectors to the lung following intra-nasal instillation ("sniffing") by the animal.[11,13] Radiotracer distribution was then assessed by gamma scintigraphy in physically partitioned head, lung, and remaining body sections, comparing the 100 µL dosing condition to equivalent total radiotracer quantities separated over multiple 5 µL intranasal pipette bolus administrations, each spaced 5 min apart ($n = 6$/group) (Figure 1A). In the single 100 µL dose cohort, radioactive liquid was distributed approximately evenly between the head, lungs, and remaining carcass at 36.1 ± 12%, 34 ± 14.3%, and 29.9 ± 4.1% of the total, respectively. This contrasts with the 2 × 5 µL cohort, in which 98.1 ± 1.2% of liquid was deposited in the head, 0.1 ± 0.2% in the lungs, and 1.8 ± 1.1% in the remaining carcass (Figure 1B).

To confirm that the radiopharmaceutical distribution was representative of dosed saline movement through the airway and was not influenced by the effect of undefined active radiotracer transport, lung tissue weights were compared between the 100 µL and 2 × 5 µL cohorts following dosing and dissection. Lungs from mice that were intranasally administered 100 µL of ^{99m}Tc-DTPA-loaded saline were, on average, 0.075 g heavier than those from the 2 × 5 µL cohort (Figure S1A, $p < 0.001$), despite the overall mouse weights (remaining carcass and head) being similar (Figure S1B; p = NS). Furthermore, for the 100 µL dose cohort, lung weight and lung radioactivity were positively correlated (Figure S1C, $p < 0.05$), suggesting that radiation measurements reflected dosed saline distribution.

With increasing numbers of small-volume applications to the nose, liquid continued to be highly restricted to the heads of mice, with an average of 94.6 ± 5.7% and 90.3 ± 2.5% of total radioactivity being measured in the heads of 5 × 5 µL and 10 × 5 µL treated mice ($n = 6$/group), respectively (Figure 1B). With all multiple small-volume administrations, significantly greater dosed volumes were retained in heads ($p < 0.001$) and were lower in lungs ($p < 0.05$) compared with the 100 µL treatment cohort. While average head liquid retention decreased as overall volumes increased, lung liquid distribution remained very low at 0.1 ± 0.2%, 0.7 ± 0.7%, and 0.3 ± 0.2% of the total in the 2 × 5, 5 × 5, and 10 × 5 groups, respectively ($n = 6$/group). In summary, these outcomes supported the development of a nose-only dosing schedule, which was next explored using lentiviral vectors to assess whether transgene expression would remain similarly spatially restricted.

Dose escalation of small-volume intranasal lentiviral vector administrations results in increased nasal tissue transgene expression with minimal lower airway biodistribution

To assess whether viral vector distribution and transgene expression were similarly influenced by multiple small-volume nose-only administrations, we compared increasing doses of rSIV.F/HN encoding a CpG-free elongation factor 1α promoter/cytomegalovirus enhancer (hCEF) driven enhanced-GFP-luciferase fusion protein, split over multiple 5 min-separated 5 µL intranasal administrations. An escalating vector dose (1e7 to 2.4e8 transducing units (TU)/mouse) and volume (2 × 5 µL up to 10 × 5 µL) was administered, and 10–12 days post dosing, firefly bioluminescence reporter transgene activity was assessed in treated and control groups by whole-body luminescence imaging ($n = 5–6$/group) (Figure 2A).

As expected, nasal transgene expression was observed following 100 µL or multiple 5 µL intranasal vector applications, with expression levels increasing with ascending total dose (Figure 2B; $p < 0.0001$). Further, in line with the outcome of the radiotracer liquid distribution study, excised lung transgene reporter signal was consistently measured only in the 100 µL treated cohort, with lung bioluminescence negative in 11 of 25 lungs from the multiple 5 µL treated mice and at an overall significantly ($p < 0.05$) lower level (Figure 2C). As an additional sensitive assay, nasal tissue from each multiple 5 µL, nose-only, treated mouse was processed after

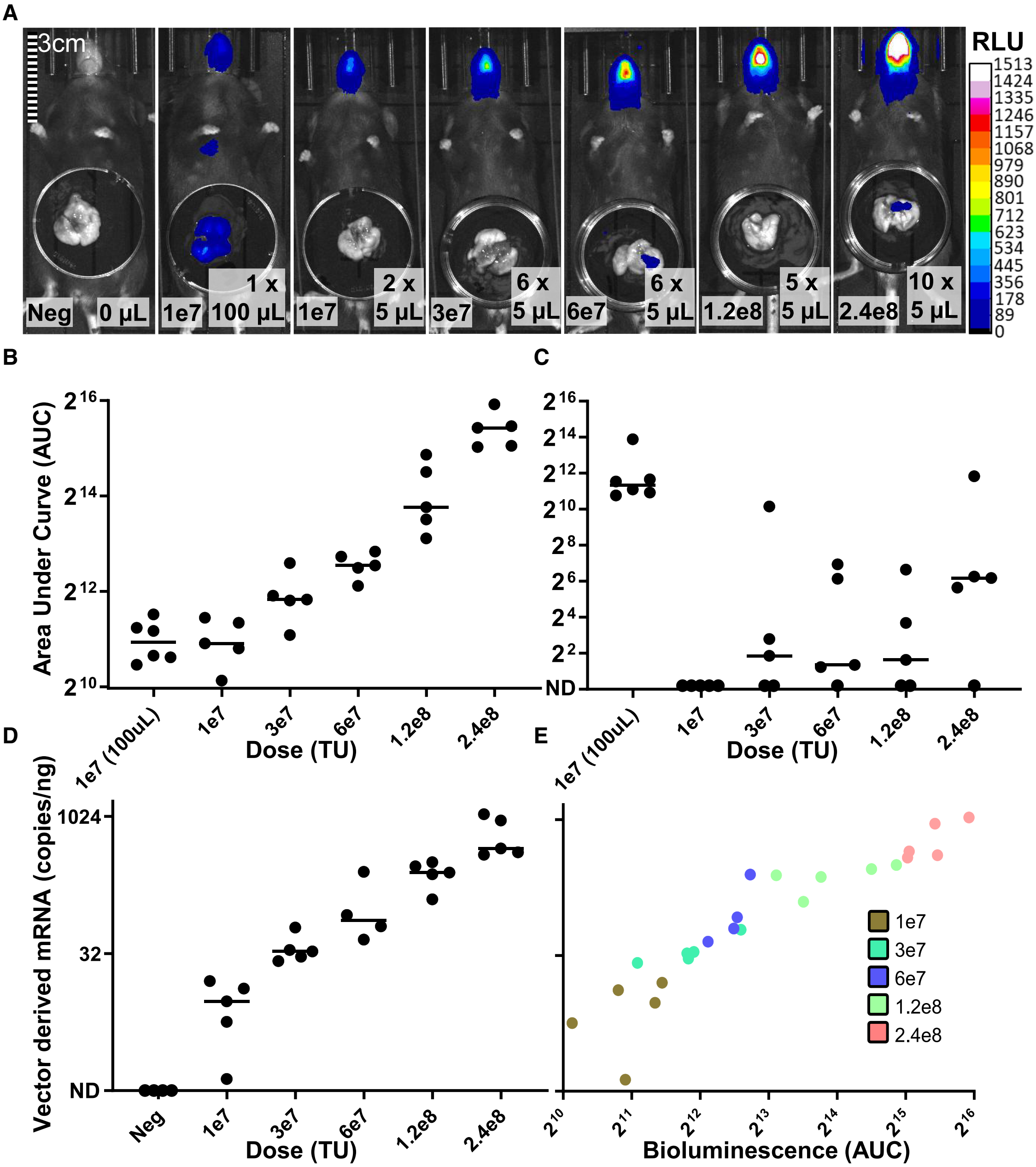

Figure 2. Transgenic protein and transcript measured in the nose and lungs of mice following intranasal administration of rSIV.F/HN

(A) Representative whole-body luciferase bioluminescence and overlaid isolated lung images from 8–12-week-old mice, comparing a single 100 μL (nose and lung targeted) application of rSIV.F/HN encoding an hCEF-driven EGFP-firefly luciferase fusion protein to multiple 5 μL (nose-only) administrations of increasing dose and overall volume. Densitometry analysis of the bioluminescence image data shows area under the curve (AUC) measurements with increasing dose (1e7-2.4e8TU) of multiple small-volume administrations, showing measurements in (B) nasal regions (Welch's ANOVA, $n = 5$–6, $p < 0.0001$: Dunnett's post-hoc test) and (C) lung tissue (ANOVA, n = 5–6, $p < 0.05$: Dunnett's post-hoc test). (D) From the same multiple small-volume treated mice and four wild-type negative controls (Neg), nasal tissue RNA viral vector WPRE transcript, expressed as transgene mRNA copies per ng of total RNA (mRNA copies/ng), was quantified by droplet digital PCR, and (E) was analyzed for correlation with bioluminescence (Spearman, r = 0.9783, $p < 0.0001$). For (B)–(D): data are represented as individual values with median. Abbreviations are as follows: RLU, relative light units; AUC, area under the curve; ND, non-detectable.

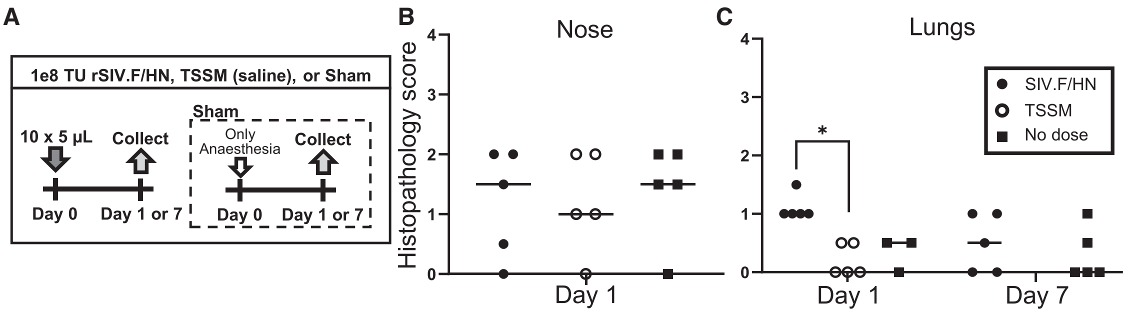

Figure 3. Mouse nasal and lung tissue histopathology tolerability study

(A) Dosing and collection timeline, comparing administration of 1e8 TU hCEF-driven EGFP-encoding lentiviral vector (rSIV.F/HN) administered in 10 aliquots of 5 μL each, separated by a 5 min interval, TSSM, or no-dose anesthesia-only (Sham) conditions. (B) Histopathology scoring of nose at day 1 (Kruskal-Wallis, $n = 5$, p = ns) and (C) lungs 1 and 7 days after vector or control treatments (Kruskal-Wallis, $p < 0.05$, Dunn's post-hoc test). Data are represented as individual values with median. * = $p < 0.05$.

bioluminescence imaging to quantify transgene mRNA levels using droplet digital PCR (ddPCR). This allowed us to confirm viral transduction and to assess whether the increased luciferase expression observed in the nose was transcript copy number-dependent across multiple small-volume administrations. Proviral-derived transgene mRNA levels, downstream of viral genetic integration into transduced cells, showed a dose-related increase across the nose-targeted administrations (Figure 2D), which was highly positively correlated with reporter protein activity from these same nasal tissues (Figure 2E, $p < 0.0001$). These data confirmed the efficacy of the multiple small-volume intranasal application strategy in containing viral transduction and transgene expression to the murine nasal epithelium and that these findings were resilient to repeated administrations and increasing overall dose. Specifically, the expression relationship remained linear over a 24-fold dose escalation, supporting the use of this lentiviral vector dosing method in subsequent studies.

No acute nasal and minimal transient lung histological changes following nose-only vector administration

We next assessed whether the nose-only administration method produced any acute toxicological changes. Tissue sections were scored for cellular pathology 1 or 7 days after treatment. A 50 μL volume, split into 10 × 5 μL volumes, each administered with a 5 min interval (total delivery time 45 min), was intranasally administered to mice to achieve a final lentiviral vector dose of 1e8 TU rSIV.F/HN, encoding an hCEF-driven enhanced GFP (n = 5/group) (Figure 3A). A similar volume of the vector diluent, TSSM (tromethamine-, sodium-, sucrose-, and mannitol-based saline), was included for the day 1 comparison only (n = 5/group). A sham-treated cohort, exposed only to isoflurane anesthesia, was also included to test for vector- and liquid-independent anesthesia effects (n = 3–5/group). In nasal tissue, there was no significant difference in total histopathology score (Figure 3B) and no evidence of inflammatory cell foci or pathologic tissue-structural features (Table S1) between vector, TSSM, or sham controls at day 1. Nasal tissue was not assessed for pathology at the day 7 time point due to the day 1 time point showing no differences.

Across lung tissue sections, overall pathological grading was low to normal but with the 10 × 5 μL lentiviral vector application there was significantly elevated grading compared with the TSSM controls at day 1 post dosing (Figure 3C; $p < 0.05$). The majority of the measured parameters were within normal limits, with the exception of a small one-increment increase in macrophage infiltrations, from normal to very limited and focal tissue infiltration, in 4 of 5 vector-treated mice compared with all TSSM controls at day 1 (Table S2). By day 7, all histopathology scores from the lungs of rSIV.F/HN-treated mice were indistinguishable from those of the control group.

Nasal tissue-targeted lentiviral vector results in long-term systemic and lung levels of a secreted protein

Administration of the rSIV.F/HN vector results in life-long pulmonary transgene expression (~2 years) in mice following inhalation of a 100 μL bolus into the lungs.[11,14] We therefore assessed whether the nose-only administration method resulted in prolonged gene expression and secreted protein detection in the lungs and the systemic circulation.

Wild-type mice were intranasally administered 10 × 5 μL aliquots of rSIV.F/HN encoding an hCEF-driven secreted reporter protein, *Gaussia* luciferase (Gluc), using the same 5 min-interspersed dosing protocol as before. This was compared with a single 100 μL intranasal or intravenous dose, with all treatments delivering a matched 2e8 TU of total vector (n = 5–7/group). Gluc activity was then quantified in serum over a subsequent 6-month period and, at study termination (6 months), in nasal and lung tissue homogenates.

High levels of reporter protein were measured at 6 months in the nose after nose-only and 100 μL bolus sniffing, but it was non-detectable following intravenous administration (Figure 4A). A similar duration of protein detection was also seen in the lungs with both nasal delivery methods, though, as expected from earlier data showing firefly luciferase (a non-secreted protein) expression (Figure 2), reporter protein levels in the lungs were lower with the nose-only method compared with the 100 μL bolus (Figure 4B) ($p < 0.001$). These data confirm the previously observed long

duration of expression in the lungs and extend these observations to secreted protein in the nose to a minimum of 6 months (the longest period assessed in this study).

We also determined whether serum levels of Gluc could be detected via either intranasal delivery method and compared this with values after intravenous delivery. At each time point studied, the optimal route for delivery into the circulation was via the 100 μL bolus dose sniffed into the lung (Figure 4C). However, interestingly, the nose-only delivery method provided systemic levels that were statistically similar to those achieved through intravenous delivery at all but one measured time point.

Correction of PAP biomarkers using nose-as-factory for GM-CSF production

We next sought to demonstrate proof-of-concept for the function of the secreted protein following the nose-only method of delivery. GM-CSF knockout mice, which recapitulate biomarkers of aPAP lung disease, were administered rSIV.F/HN hCEF vector encoding murine GM-CSF cDNA either as a single 100 μL bolus (2.3e8 TU/mouse) or using 10 (2.3e8 TU/mouse) or 5 (6e7 TU/mouse) 5 μL aliquots via nose-only delivery (n = 6–8/group). Negative control mice remained untreated (n = 15).

GM-CSF toxicity is well recognized[15,16] and, in keeping with this, only the 6e7 TU mice could be assessed at the intended end of study (2 months post-dosing). At the higher (2.3e8 TU/mouse) dose, animal husbandry issues in the 100 μL bolus cohort limited the assessment period to 2.5 weeks post-dosing, and the 10 × 5 μL treated mice (equivalent dose of 2.3e8 TU/mouse) were therefore simultaneously collected at this earlier matched time point. Specifically, in the 100 μL administration group only, 2 of the 8 treated mice died prematurely, with the remaining 6 mice displaying symptoms of distress, including piloerection and reduced exploratory behavior.

At 2 months post-treatment, bronchoalveolar lavage fluid (BALF) GM-CSF levels averaged 0.1 ± 0.1 ng/mL in the low-dose nose-only (5 × 5 μL) cohort; two of nine samples fell just below the assay standard range of linear quantification, and levels were not different from those in untreated control mice (Figure 5A). BALF turbidity is a measure of aPAP-associated lung surfactant and, in keeping with the GM-CSF levels, did not differ between these two groups (Figure 5B). Finally, pulmonary alveolar surfactant (PAS) lung tissue staining was lower in low-dose nose-only mice compared with untreated aPAP controls ($p < 0.05$), indicative of a degree of aPAP lung disease phenotype correction following lentiviral vector application (Figure 5C).

At 2.5 weeks post-treatment, the high-dose cohorts showed average lung GM-CSF levels of 12.4 ± 8.5 ng/mL (10 × 5 μL) and 38.2 ± 19.6 ng/mL (100 μL bolus), not significantly different from each other, but with the 100 μL cohort significantly greater than the no-treatment control ($p < 0.0001$; Figure 5A). Consistent with the higher levels of lung GM-CSF in these animals, both BALF turbidity (Figure 5B) and tissue PAS staining (Figure 5C) were significantly lower than those in no-treatment aPAP model mice ($p < 0.05$), again indicating lung disease amelioration. Consistent with our previously published observations, PAS was the more sensitive disease marker post-vector dosing.

Thus, the nose-only route is able to produce sufficient GM-CSF to ameliorate lung disease, both at 2.5 weeks and 2 months post-dosing. However, GM-CSF stimulates proliferation and colony formation of macrophages and, therefore, may induce toxicity, as has been previously described. Consistent with such macrophage accumulation, significant lung tissue consolidation was measured using a total area-normalized image analysis approach (Figure 5D). In animals treated with the lower nose-only dose of 6e7 TU of vector, no tissue consolidation above no-treatment aPAP baseline levels was observed in lungs at the 2-month time point, indicating that dose titration was achieved, resulting in lung disease correction with negligible toxicity (Figure 5E). However, in animals receiving the higher nose-only dose of 2.3e8 TU of vector, lung tissue consolidation was increased at the 2.5-week time point compared with untreated controls ($p < 0.005$). The highest average level of lung tissue consolidation was measured following the 2.3e8 TU dose of rSIV.F/HN delivered via the 100 μL bolus, which was significantly greater than both untreated ($p < 0.0001$) and 6e7 TU/mouse nose-only treated mice ($p < 0.005$), consistent with the higher lung levels of GM-CSF in this cohort. The link between lung GM-CSF level and consolidation was further underlined by a significant positive correlation between both metrics in the 100 and 10 × 5 μL dosing groups (Figures S2A and S2B), reinforcing the benefit of limiting secreted transgene protein amounts in the lungs to only therapeutic levels via the nose-only application strategy.

DISCUSSION

We describe a method to restrict liquid dosing to the murine nose, demonstrate safety and duration of efficacy using a lentiviral vector, and provide proof-of-concept that the resultant protein is functional and can correct phenotypic changes in a murine model of lung disease. Specifically, we show that intranasally administered viral vector instillation volume affects its distribution and transgene expression *in vivo*. Multiple small volumes were optimal in restricting vector deposition and transgene expression to the nose but still resulted in effective lung and systemic availability for secreted proteins. These data may help catalyze the use of the human nose as a factory for the production of therapeutic proteins needed in the nose, lung, or indeed systemic circulation.

Using a Tc radiotracer study, we assessed retention of the delivered volume within the nose or lung. At a high volume (100 μL), approximately one-third reached either site, with the remaining one-third assumed to be primarily swallowed. Southam et al.[17] showed that a single 50 μL bolus intranasally administered to mice resulted in 55.7 ± 2.5% of instilled liquid reaching the lungs. In comparison, here, by separating the same 50 μL total volume into multiple 5 μL aliquots, only 0.3 ± 0.2% of the total volume reached the lungs. The data suggest that with larger volumes, the inspiratory respiratory

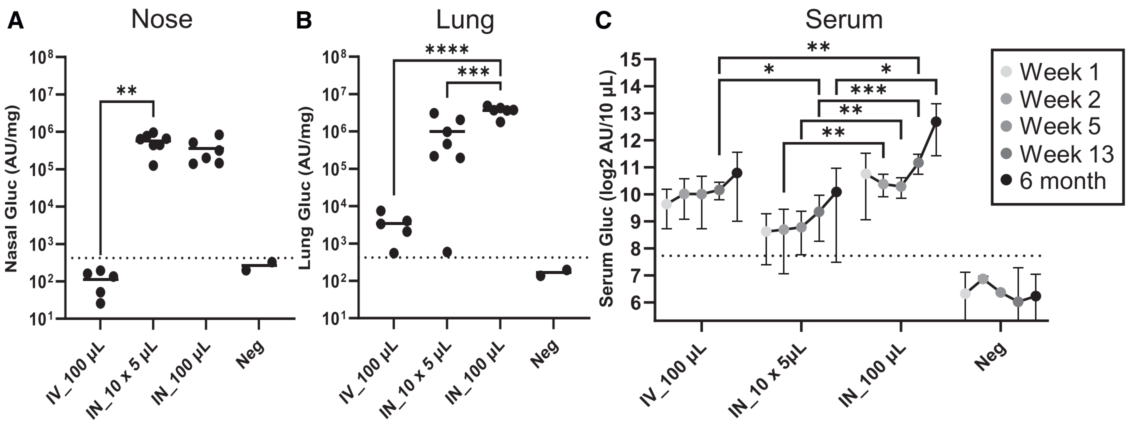

Figure 4. *Gaussia* luciferase protein activity measured in nose and lung tissue homogenates and serum over 6 months

Gluc activity was measured following nose-only 10 × 5 μL bolus dosing (IN_10 × 5 μL), a single 100 μL bolus intranasal (IN_100 μL), or 100 μL intravenous (IV_100 μL) administration of 2e8TU rSIV.F/HN lentiviral vector, or untreated controls (Neg). Total protein-normalized (per mg) Gluc activity was measured in (A) nose (ANOVA, $n = 5$–7, $p < 0.05$; Tukey's post hoc test) and (B) lung (ANOVA, $n = 5$–7, $p < 0.0001$; Tukey's post hoc test) tissue homogenates (data are represented as individual values with mean). (C) Teil-vein derived blood serum measurements at multiple time points from the same mice (two-way ANOVA, $n = 5$–7, $p < 0.0001$; Tukey's post-hoc test) (data are represented as mean ± SD). Samples from two wild-type untreated mice (Neg) were included for assigning the assay negative thresholds (dotted lines = negative average + [3 × standard deviation]) and were not included in any statistical tests. AU, arbitrary units. * = $p < 0.05$; ** = $p < 0.005$; *** = $p < 0.0005$; **** = $p < 0.0001$.

effort ("sniffing") of the mice is sufficient to draw liquid into the lungs, but this is minimized at the smaller 5 μL volumes studied here. This volume is also consistent with the sub ∼7.5 μL volumes of an iodine-based contrast fluid shown to be retained in the mouse nose, reported by Donnelley et al.[18] Taking this further, we describe a repeat administration protocol, increasing the total liquid or viral vector administered while maintaining only minimal liquid or vector spillover into the lungs. Given the ability of humans to mouth breath (not present in mice), involuntary lung administration following nasal delivery is unlikely to be a problem when translating this methodology. Also relevant here is the eventual mode of product delivery to the human nasal epithelium. There are a range of devices already developed for liquid-based drug delivery to the nasal mucosa in humans, in most cases designed to limit per-unit volume or droplet sizes to those best suited for upper airway deposition.[19] A re-interrogation of such devices was not an aim of the present study, which instead sought to validate the overall viral vector intranasal delivery strategy from a biophysical and biological perspective by overcoming the experimental hurdle of nasal delivery in rodent models. For eventual human testing, another relevant factor that we could not model here is human adult versus juvenile nasal tissue targeting. Others have shown that lung deposition of inhaled drugs tend to be higher in adults compared with children and that the outcome of adult studies should not be extrapolated for use in children when assessing inhaled formulations.[20,21] We anticipate that this would be similar for nasal delivery of our viral vector and that required volumes may not scale linearly with person weight or even respiratory surface area, in line with the above literature observations. Similarly, it is difficult to predict whether human male or female respiratory anatomical differences,[22] such as a height-matched reduction in airway lumen area size in females,[23] will have a significant impact on viral vector-induced gene expression. In all cases, it will be important to carefully design clinical trials to account for potential age- and sex-related factors, which was not the direct focus of the present study.

Delivering a gene encoding a non-secreted reporter protein, we confirmed mostly head-restricted transgene expression following the small-volume intranasal application strategy. We did not address the possibility of extra-mucosal brain and central nervous system viral vector exposure and transduction in the present study, but we have measured non-target organ transduction as part of another study (Bell et al., manuscript under review) and did not find evidence of brain transduction there.

Interestingly, when assessed at 6 months following nose-only delivery, expression of Gluc was similar in nose and lungs, and we further saw a time-related increase in levels of this secreted protein in the circulation. We assume the most likely explanation is continuous secretion of Gluc from the vascular nasal epithelium, with a concomitant build-up of levels in the circulation, which then spills into the lungs. We cannot exclude a contribution of low-level lung transduction following nose-only delivery, since we did not quantify lung transduction directly here, and we did confirm low-level lung transgene expression following nose-only delivery for the non-secreted reporter protein previously (Figure 2). However, based on the very low levels of apparent lung transduction in the non-secreted reporter experiment, coupled with radiotracer outcomes suggesting equally low liquid distribution to the lungs following small-volume inhalation, it seems reasonable to hypothesize a much greater influence of systemic circulation on lung protein spillover in this scenario. Independent of this factor, while circulatory levels of the transgenic

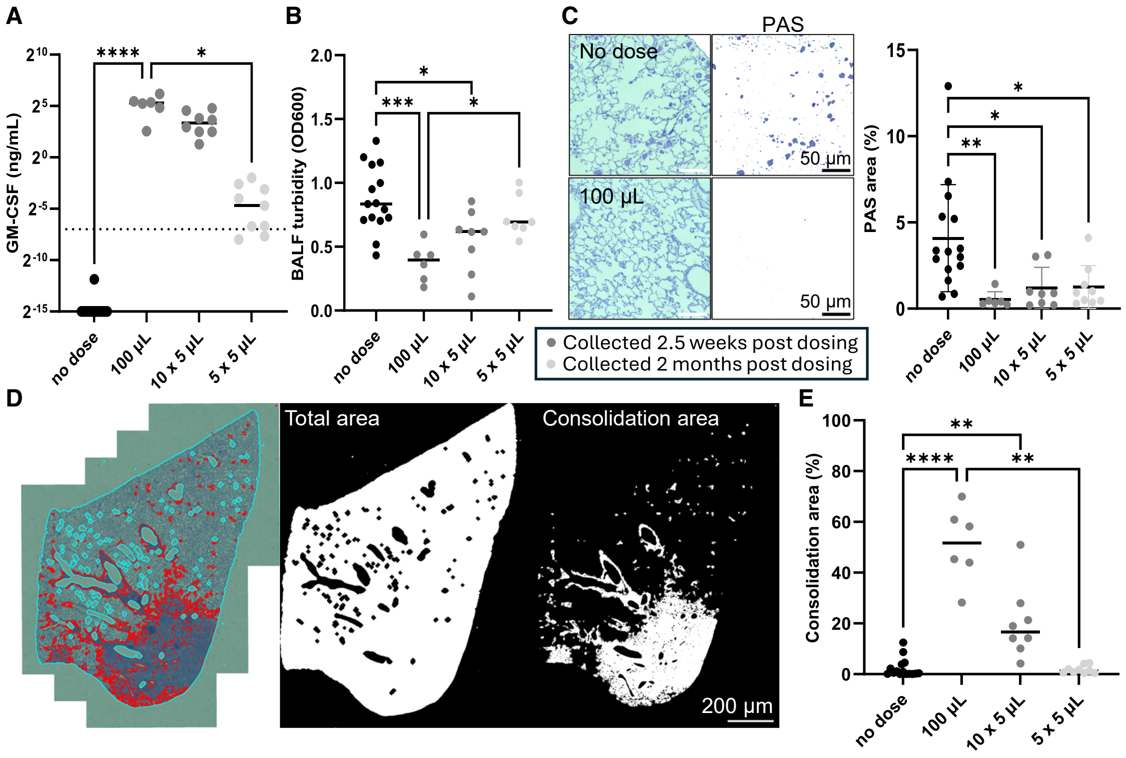

Figure 5. GM-CSF transgene and aPAP mouse phenotype lung-level quantification at 2.5 weeks (100 μL and 10 × 5 μL treated mice given 2.3e8TU vector) and 2 months (5 × 5 μL treated mice given 6e7TU vector) post intranasal rSIV.F/HN treatment

(A) Bronchoalveolar lavage fluid (BALF) GM-CSF levels measured by ELISA (Kruskal-Wallis, n = 6–15, $p < 0.0001$: Dunn's post-hoc test) (data are represented as individual values with median; dotted line = lowest experimental standard of 7.8 pg/mL). (B) BALF turbidity measured as optical density (OD: 600 nM) (ANOVA, n = 6–15, $p < 0.0005$: Tukey's post-hoc test) (data are represented as individual values with median). (C) Pulmonary alveolar surfactant (PAS) staining example images from no-dose and 100 μL bolus-treated mouse lungs (left) and image analysis quantification of all left-lung lobes (right) (Welch's ANOVA, n = 6–15, $p < 0.005$: Dunnett's post-hoc test) (data are represented as individual values with mean ± SD). (D) Example images showing how lung consolidation was measured from PAS-stained whole left-lung coronal sections (left), using image analysis to measure whole-lung area (middle) and consolidation area (right). (E) Consolidation area, as a percentage of total tissue area, plotted across all left-lung samples (Kruskal-Wallis, n = 6–15, $p < 0.0001$: Dunn's post-hoc test) (data are represented as individual values with median). * = $p < 0.05$; ** = $p < 0.005$; *** = $p < 0.0005$; **** = $p < 0.0001$.

protein following nose-only delivery were not as high as those produced by either a single nasal bolus dose or following direct intravenous delivery, they were sufficient to ameliorate the pulmonary manifestations of PAP in subsequent GM-CSF testing. Specifically, histological assessment of lung tissue showed lower levels of PAS in all vector-treated PAP mice, including the nose-only cohorts, compared with untreated controls. That statistical significance was not achieved for BALF turbidity in the low-dose nose-only group compared with untreated controls is also consistent with our experience of histological lung surfactant measurement being the more sensitive biomarker for mouse disease assessment.[16] Altogether, this suggests that the nose-only route may produce sufficient levels of systemic proteins for therapeutic benefit, potentially opening up this delivery route for the treatment of conditions such as the haemophilias, thrombotic thrombocytopaenic purpura, and others.

Our data may present an interesting opportunity to easily titrate secreted proteins and match these levels to defined therapeutic efficacy windows. As a proof-of-concept, we show that nose-targeted expression of GM-CSF resulted in sufficient lung levels of the therapeutic protein to improve the lung surfactant phenotype of PAP, while limiting the toxicity linked to a lung-targeted delivery route. Since dose-outcome relationships are generally variable between individuals, influenced by innumerable known and unknown biological, physical, and experimental factors,[24–26] this type of individually tailored dosing may be helpful, particularly in the context of either known vector or therapeutic protein toxicity. The PAP mouse model was especially useful for testing this form of dose titration, since only low levels of lung GM-CSF, often below the normal limit of commercial ELISA quantification, are required to prevent PAP, while the cytokine's lung and systemic toxicity at only moderately higher levels are also well noted.[16,27–29] Furthermore, the human nasal cavity exhibits greater immune tolerance to exogenous biologics than lower respiratory sites or most other body compartments,[30] especially without adjuvant co-administration.[31] In some cases, IgG or IgM binding and more pro-inflammatory immune response states are

suppressed in the nasal cavity via mechanisms such as IgA coating[9,32] and, finally, it is a highly accessible tissue with a surface area of 150–200 cm^2.[33] For the current study, we did not attempt to define all aspects of vector-related immunogenicity or systemic toxicity, instead choosing to focus on acute nose and lung tissue-level histopathology, in line with the dosing strategy agenda of our work. Even for the GM-CSF study, we limited toxicity assessment to only lung consolidation associated with transgenic protein levels in that tissue. A rationale for the more limited toxicity assessment here is that, in previous work, we have already shown that 100 μL nose-and-lung delivery of rSIV.F/HN encoding a reporter protein did not result in vector-related extra-pulmonary spleen, liver, or kidney measures of cellular pathology over baseline controls.[16] Additionally, in a recent non-human primate safety assessment of our vector, we noted no significant body weight, organ weight, or broad-panel cytokine level changes in vector- versus diluent-treated animals 7 days after treatment.[34] In line with our earlier statements, we acknowledge that a nose-only administration of our viral vector may result in a different immunological outcome compared with a lung- and nose-delivered product, but this is something we did not experimentally explore here.

Further, there are implications for the volume of vector that is required to be manufactured using a nose-only delivery strategy, potentially limiting the cost of goods for these advanced therapy products. Applications of lentiviral vector to this site in adult humans may include future treatments for alpha-1 antitrypsin deficiency,[1] genetic surfactant deficiencies, and the many disorders for which antibody treatments are viable,[35] including against infectious pathogens such as influenza[36,37] or severe acute respiratory syndrome coronavirus 2 (SARS-CoV-2).[38]

An alternative approach is to ask whether therapeutic expression is required in the nose, lung, or systemic circulation, and which is the optimal delivery site. Our data suggest that, to obtain expression in the nose, localizing delivery to that organ via topical delivery is considerably superior to the intravenous route. For expression in the lung, nasal or pulmonary delivery are approximately equivalent (when matched for dose deposited) and both are superior to the intravenous route. Finally, for expression in the serum, pulmonary delivery is optimal, with significant levels obtainable via the intranasal route. If these data translate to humans, they may suggest alternative delivery approaches for the treatment of multiple conditions.

In conclusion, our data suggest the nasal cavity is both an effective site for delivery of vectors engineered to secrete therapeutic proteins and a simple, likely translatable way that this might be achieved.

MATERIALS AND METHODS

99mTc-DTPA radiotracer study

All animal studies were approved by the Imperial College Animal Ethics Committee and carried out according to Home Office regulations and the Animal Research: Reporting of *In Vivo* Experiments (ARRIVE) guidelines, as appropriate. Female C57BL/6N mice of 8–12 weeks of age were used. A single 100 μL or multiple 5 μL volume(s) of ^{99m}Tc-DTPA (5 MBq in PBS) were administered to the mouse nares. Clinical-grade ^{99m}Tc-DTPA was obtained from St. Bartholomew's Hospital radiopharmacy (St Bartholomew's Hospital, West Smithfield, City of London, EC1A 7BE). Delivery was secured for the day of each experiment. Radioactivity of the delivered product was confirmed using a gamma counter, and the activity was then diluted in PBS to obtain the desired radioactivity per unit volume. Previous studies have shown that a 100 μL volume is rapidly sniffed into the lungs as a standard delivery method.[6,11,16] Multiple volumes were administered with a 5-min interval between each. ^{99m}Tc-DTPA is a common clinical radiopharmaceutical used in human pulmonary and gastric diagnostic studies. There is no evidence that the molecule is actively transported toward, nor selectively taken up by, specific organs or tissues. Dosing procedures were conducted in mice under isoflurane inhalation anesthesia (2%–3% maintenance), with animals briefly exposed to normal air only for the duration of each administration. Mice were maintained under anesthesia for 10 min following their single or final ^{99m}Tc-DTPA dose, after which they were culled by cervical dislocation (with carotid artery severance as a confirmation).

Radioactivity was measured in the dissected head, lungs, and remaining carcass on the day of dosing (1–2 h after death) and on the following day (24–28 h after death), using a 2470 Wizard-2 gamma counter (PerkinElmer, Beaconsfield UK). Reported data are from the second measurement in all cases, as the total dose of 5 MBq resulted in counts exceeding the Wizard-2 maximum count rate of approximately 5,000,000 counts-per-minute (CPM) when measured on the day of the dissection.

Lentiviral vector production and quantification

All lentiviral vectors used in this study were manufactured by the Gene Medicine Research Group, University of Oxford. Suspension-cultured HEK293T cells were co-transfected for rSIV.F/HN production, then vector was purified using anion-exchange chromatography, concentrated using tangential flow filtration, and assessed for functional titer, all as previously described.[14]

rSIV.F/HN dosing

rSIV.F/HN lentiviral vector expressing EGFP and firefly luciferase (vGM020: rSIV-hCEF-EGFPlux2), driven by a CpG-free hCEF,[39] was used in dose escalation and bioluminescence lentiviral vector experiments. Female C57BL/6N mice (Charles River Laboratories, Margate, UK) of 8–12 weeks of age were isoflurane anesthetized (2%–3% maintenance) and transduced with set volumes of rSIV.F/HN by nasal administration. Viral vector was diluted in TSSM (20 mM Tris, 100 mM NaCl, 10 mg/mL sucrose, 10 mg/mL mannitol, pH 7.3) vehicle solution prior to nasal administration to achieve the target total doses. Bioluminescence imaging and tissue collections were completed 10–12 days following dosing (see below).

rSIV.F/HN lentiviral vector expressing EGFP (vGM196: rSIV-hCEF-EGFP) was used in lentiviral vector tolerability experiments. In this study arm, female C57BL/6N mice of 8–10 weeks of age were

isoflurane anesthetized and intranasally administered set volumes of viral vector were diluted in TSSM buffer prior to nasal administration to achieve the target total doses. Nasal and lung tissue collections were undertaken 1 or 7 days following dosing. No-treatment control mice were anesthetized for approximately 45–50 min with isoflurane to mimic intranasal treatment. They were also periodically handled while under anesthesia to further mimic the treated group.

rSIV.F/HN lentiviral vector expressing codon-optimized Gluc (vGM124: rSIV.F/HN-hCEF-soGluc) was used in the 6-month secreted transgene tracking experiments. In this study arm, male and female C57BL/6N mice of 8–10 weeks of age were intranasally or intravenously treated with viral vector diluted in TSSM buffer to achieve the target total doses. Intranasal delivery was administered as either a single 100 μL volume or as 10 × 5 μL applications. A 100 μL volume was used for each intravenous administration. Blood collections, luminescence measurements, and tissue collections were undertaken during and after the 6-month period following dosing (see below).

rSIV.F/HN lentiviral vector expressing mouse codon-optimized GM-CSF (vGM173: rSIV.F/HN-hCEF-msoGMCSF) was used in the PAP treatment study. In this study arm, male and female GM-CSF knockout mice (B6.129S-Csf2^{tm1Mlg}/J, Jackson Laboratory, Bar Harbor, Maine, USA) of approximately 6 months of age were intranasally administered viral vector diluted in TSSM to achieve target total doses. Dosing volumes were applied as either a single 100 μL bolus or as 10 × 5 or 5 × 5 μL applications.

Nose and lung histopathology preparation and assessment

At 1 or 7 days following treatment, mice were culled by cervical dislocation (with carotid artery exsanguination for confirmation). Lung and nasal tissues were then collected and processed as described below.

a) Lungs. A needle-guided cannula (Abbocath-T 22G x 25 mm, BD UK) was used to intubate the mouse trachea before syringe inflation of the lungs with 4%$^{w}/_{v}$ paraformaldehyde (PFA: pH7.4, in PBS). Inflated lungs were then submerged in the same fixative for a 24 h incubation at 4°C. Following this fixation period, lungs were placed into a 70%$^{v}/_{v}$ ethanol solution, ready for paraffin wax embedding and sectioning. Wax-embedded lungs were sectioned on a microtome (Leica RM2125) at 6 transverse levels through the tissue (2 mm between levels), with a section thickness of 5 μm.
b) Nose. The mouse nose and head were surgically cleared of fur, external soft tissues, the lower jaw, and portions of the head posterior to the eye sockets, before being placed into 4%$^{w}/_{v}$ PFA (pH 7.4, in PBS) for a 24 h incubation at 4°C. The nose was then transferred to a 0.45 μm syringe-filtered 20%$^{w}/_{v}$ EDTA solution (pH 7.4, in distilled water) for a prolonged incubation with orbital shaking to decalcify the bones. The nose was incubated in the EDTA solution for 10 days, with changes to fresh 20% EDTA every 2 days. Following decalcification, the nose was thoroughly washed in running tap water for several minutes and then transferred to 70%$^{v}/_{v}$ ethanol (in distilled water), ready for wax embedding and sectioning. Wax-embedded snouts were sectioned on a microtome (Leica RM2125) at 5 transverse levels through the tissue (1 mm between levels), with a section thickness of 5 μm.

Sectioned tissue from all collected lung and nose levels was stained with haematoxylin and eosin using a semi-automated platform (Leica ST5010 AutoStainer XL), ready for sample-blinded pathology scoring undertaken by a veterinary histopathologist (Central Diagnostic Services, Department of Veterinary Medicine, University of Cambridge, UK). Pathology scoring was completed in alignment with the recommendations of Mann et al.,[40] by which cellular and structural features of pathology were assessed for their local and whole-section severity and distribution in a compound model analysis, with a summative semi-quantitative grade assigned on a scale of 0 (within normal limits) to 4 (severe).

Bioluminescence measurement and analysis

Luciferase SIV.F/HN transgene expression was measured using both the IVIS Lumina III series (PerkinElmer, Beaconsfield UK) imager for whole mice or isolated whole tissues and a FLUOstar Omega microplate reader (BMGlabtech, Aylesbury UK) for tissue homogenates and blood samples.

Whole mouse/tissue measurements

With the mouse under isoflurane anesthesia, 50 μL of VivoGlo D-luciferin (37 mg/mL in PBS [-Ca2+, -Mg2+]) were administered to the nares for "sniffing," 10 min before imaging. Whole-body *in vivo* firefly bioluminescence was measured using a 1 min exposure time, with the mouse under continual isoflurane anesthesia. The mouse was then culled by cervical dislocation (with carotid artery severance as confirmation) for lung excision and separate bioluminescence imaging. One-half of the lung tissue was then snap-frozen in dry ice for protein extraction, and the other half processed for RNA extraction (see below). Lung halves for homogenization were collected and frozen directly into FastPrep Lysing Matrix D 2 mL tubes (MP Biomedicals, Irvine, CA, USA). Luminescence relative light units (RLU) were determined by densitometry analysis of constant region-of-interest (ROI) areas of the IVIS 16-bit grayscale capture images, using ImageJ.[41]

Microplate tissue-homogenate measurements

If not collected directly into the tube, frozen tissue was moved into a FastPrep Lysing Matrix D 2 mL tube. The frozen lung half was placed on ice and allowed to thaw briefly. While still barely frozen, 500 μL of 1x Reporter Lysis Buffer (Promega, Southampton UK) was added to the tissue, and the sample was homogenized using a FastPrep-24 homogenizer (MP Biomedicals, Irvine, CA, USA) for up to 1 min. Lysis Matrix tubes containing homogenized sample were then directly centrifuged at 14,000 × g for 15 min at 4°C. Protein supernatant was stored at −80°C or, preferably, processed immediately. 20 μL of each homogenized protein sample was added to individual wells of a non-transparent 96-well plate. For firefly luciferase activity

measurements, 100 μL of 1× Luciferase Assay reagent (Promega, Southampton UK) was automatically injected into each well before measurement. For Gluc measurements, 50 μL of 1× Luciferase Glow Assay reagent (Thermo Fisher Scientific, Cheshire UK) was automatically injected into each well before measurement. Total protein quantification, required for luminescence normalization, was performed using a portion of the sample protein homogenate in a DC protein assay (Bio-Rad, Watford UK).

Gluc measurements from blood serum

At set periods (1, 2, 5, 13, and 24 weeks) after viral vector dosing (see above), approximately 50–100 μL of tail vein blood was collected from mice via an open venepuncture method, using a 25G needle. Blood was stored on ice during coagulation and transport to a laboratory, then centrifuged at 3,000 × g for 10 min at 4°C. 10 μL of the serum supernatant was added to individual wells of a non-transparent 96-well plate. 50 μL of 1× Luciferase Glow Assay reagent was automatically injected into each well before measurement.

Transgene copy number quantification

RNA extraction

All nasal tissue was dissected following IVIS bioluminescence imaging. Samples were placed into RNAlater (Thermo Fisher Scientific, UK), then stored at 4°C. Within 1 month of collection, samples were homogenized in Lysis Matrix tubes containing RLT buffer (1% $^{v}/_{v}$ 2-mercaptoethanol) and RNA was extracted using an RNeasy Mini Kit (Qiagen, Manchester UK). RNA was stored at −80°C, ready for one-step ddRT-PCR.

ddRT-PCR

Approximately 10 ng of sample RNA was used in a one-step ddRT-PCR reaction (Bio-Rad, Watford UK), using primers and a FAM probe against the woodchuck hepatitis virus posttranscriptional element (WPRE) of the lentiviral vector genome. Reaction droplets were generated, followed by RT-PCR: 50°C for 1 h, 95°C for 10 min; followed by 40 cycles of 95°C for 30 s and 60°C for 1 min; ending with 98°C for 10 min and a 4°C hold. Droplets were analyzed using the QX200 Droplet Reader (Bio-Rad, Watford UK). Assay-derived DNA copy numbers per unit volume was used as a 1:1 proxy for mRNA template, and the final readout was normalized to input RNA to yield WPRE copies/ng RNA. WPRE-Fv: 5′-CCCGGAAAGGAGCTGACA-3′; WPRE-Rv: 5′-TGGCGTGGTGTGCACTGT-3′; WPRE-probe: 5′-/5FAM/TTGCTGACG/ZEN/CAACCCCCACTGG/3IABkFQ/-3′.

GM-CSF quantification and assessment of aPAP biomarkers

Mouse GM-CSF protein was measured by Quantikine ELISA (R&D Systems, Abingdon UK) from BALF. BALF was collected using a needle-guided cannula (Abbocath-T 22G x 25 mm, BD UK) to intubate the mouse trachea, before 2–3 lavage flushes of the lungs with 1 mL of PBS. BALF was stored at −80°C if not used immediately.

BALF turbidity was measured by photoabsorbance at 600 nm using a FLUOstar Omega microplate reader (BMGlabtech, Aylesbury UK). Periodic acid-Schiff staining was applied to transverse microtome lung sections, prepared as above from formalin-fixed, paraffin-embedded tissues. Whole cross-section brightfield images of lungs were captured using a Zeiss Axio Observer (Zeiss, Cambourne UK) widefield microscope with LED illumination and a fully motorized stage. Image analysis was performed using a custom color deconvolution macro (“PAS_macro_02-10-24”) in ImageJ,[41] quantifying staining area as a percentage of total lung tissue area, excluding any areas of consolidation in the analysis. Consolidation area was measured separately, employing another color deconvolution-based macro (“Nuclei-consolidation_macro_07-10-24”).

Statistical analysis

All statistical tests were performed to compare independent groups of $n \geq 5$, with each biological replicate derived from a separate mouse in all cases (except for repeat blood collections, which were accounted for separately). The Student *t* test was used when analyzing normally distributed parametric data of approximate equal variance when comparing only two groups. When comparing a single response variable across three or more groups in the same analysis, one-way ANOVA (with Tukey’s post-hoc) was used when data adhered to parametric assumptions, and the Kruskal-Wallis (with Dunn’s post-hoc test) was used when both equal variance and data normality could not be confirmed by either the Shapiro-Wilk or Browne-Forsythe tests, respectively. In cases where data normality was confirmed but equal variance was violated, Welch’s one-way ANOVA (with Dunnett’s post-hoc test) was used. When analyzing data including two response variables (e.g., time and protein activity), two-way ANOVA (with Tukey’s post-hoc test) was used, including parameterization for repeated measures since samples were collected from the same animals at different time points. All data plotting and analysis were completed using GraphPad Prism software (v10.4.1). In all cases, the null hypothesis was rejected at $p < 0.05$; means or medians are presented on plots as specified; and standard deviations are reported following ± in the main text.

DATA AND CODE AVAILABILITY

The datasets generated and analyzed for the current study are available from the corresponding author on reasonable request.

ACKNOWLEDGMENTS

This research was funded through the Wellcome Trust Health Innovation Fund and with support from the Department of Health & Social Care and Wellcome Trust through the Health Innovation Challenge (HIC) Fund.

This publication presents independent research supported by the Health Innovation Challenge Fund (106878/C/15/Z and HCIF-R10-698), a parallel funding partnership between the Department of Health and Wellcome Trust. The views expressed in this publication are those of the authors and not necessarily those of the Department of Health or Wellcome Trust.

The Technetium radiotracer study was part-funded by Boehringer Ingelheim International GmbH. The authors greatly appreciate access to equipment and specialist support at the National Heart and Lung Institute, Imperial College London Facility for Imaging by Light Microscopy (FILM) and the Faculty of Medicine Biological Imaging Center. EWFWA is an NIHR Emeritus Senior Investigator.

AUTHOR CONTRIBUTIONS

Conception of the study, E.W.F.W.A., A.S., and U.G.; design of the work, U.G., E.W.F.W.A., D.R.G., S.C.H., and A.S.; data acquisition, A.S., R.B., C.I.J.-M., C.M., M.A.V., and E.C.; data analysis, A.S.; interpretation of data, U.G., E.W.F.W.A., and A.S.

DECLARATION OF INTERESTS

E.W.F.W.A is a consultant and Advisory Board member, Boehringer Ingelheim, and Founder Director and Consultant, AlveoGene Ltd. U.G. is a co-founder and consultant for AlveoGene Ltd, a consultant for Boehringer Ingelheim, and a non-executive director of the Cell and Gene Therapy Catapult. The authors have a patent application related to this work.

SUPPLEMENTAL INFORMATION

Supplemental information can be found online at https://doi.org/10.1016/j.omta.2026.201733.

REFERENCES

1. Chiuchiolo, M.J., and Crystal, R.G. (2016). Gene Therapy for Alpha-1 Antitrypsin Deficiency Lung Disease. Ann. Am. Thorac. Soc. *13*, S352–S369.
2. Mahlangu, J., Kaczmarek, R., von Drygalski, A., Shapiro, S., Chou, S.C., Ozelo, M.C., Kenet, G., Peyvandi, F., Wang, M., Madan, B., et al. (2023). Two-Year Outcomes of Valoctocogene Roxaparvovec Therapy for Hemophilia A. N. Engl. J. Med. *388*, 694–705.
3. Pipe, S.W., Leebeek, F.W.G., Recht, M., Key, N.S., Castaman, G., Miesbach, W., Lattimore, S., Peerlinck, K., Van der Valk, P., Coppens, M., et al. (2023). Gene Therapy with Etranacogene Dezaparvovec for Hemophilia B. N. Engl. J. Med. *388*, 706–718.
4. Auricchio, A., O'Connor, E., Weiner, D., Gao, G.P., Hildinger, M., Wang, L., Calcedo, R., and Wilson, J.M. (2002). Noninvasive gene transfer to the lung for systemic delivery of therapeutic proteins. J. Clin. Investig. *110*, 499–504.
5. Hervé, V., Rabbe, N., Guilleminault, L., Paul, F., Schlick, L., Azzopardi, N., Duruisseaux, M., Fouquenet, D., Montharu, J., Redini, F., et al. (2014). VEGF neutralizing aerosol therapy in primary pulmonary adenocarcinoma with K-ras activating-mutations. mAbs *6*, 1638–1648.
6. Paul-Smith, M.C., Pytel, K.M., Gelinas, J.F., McIntosh, J., Pringle, I., Davies, L., Chan, M., Meng, C., Bell, R., Cammack, L., et al. (2018). The murine lung as a factory to produce secreted intrapulmonary and circulatory proteins. Gene Ther. *25*, 345–358.
7. Shrewsbury, S.B. (2023). The Pharmacokinetics of Drugs Delivered to the Upper Nasal Space. Pharm. Med. (Lond.) *37*, 451–461.
8. Griesenbach, U., Cassady, R.L., Ferrari, S., Fukumura, M., Müller, C., Schmitt, E., Zhu, J., Jeffery, P.K., Nagai, Y., Geddes, D.M., et al. (2002). The nasal epithelium as a factory for systemic protein delivery. Mol. Ther. *5*, 98–103.
9. Travis, C.R. (2020). As Plain as the Nose on Your Face: The Case for A Nasal (Mucosal) Route of Vaccine Administration for Covid-19 Disease Prevention. Front. Immunol. *11*, 591897.
10. Alton, E.W.F.W., Beekman, J.M., Boyd, A.C., Brand, J., Carlon, M.S., Connolly, M.M., Chan, M., Conlon, S., Davidson, H.E., Davies, J.C., et al. (2017). Preparation for a first-in-man lentivirus trial in patients with cystic fibrosis. Thorax *72*, 137–147.
11. Griesenbach, U., Inoue, M., Meng, C., Farley, R., Chan, M., Newman, N.K., Brum, A., You, J., Kerton, A., Shoemark, A., et al. (2012). Assessment of F/HN-pseudotyped lentivirus as a clinically relevant vector for lung gene therapy. Am. J. Respir. Crit. Care Med. *186*, 846–856.
12. Davies, J.C., Polineni, D., Boyd, A.C., Donaldson, S., Gill, D.R., Griesenbach, U., Hyde, S.C., Jain, R., McLachlan, G., Mall, M.A., and Alton, E.W.F.W. (2024). Lentiviral Gene Therapy for Cystic Fibrosis: A Promising Approach and First-in-Human Trial. Am. J. Respir. Crit. Care Med. *210*, 1398–1408.
13. Mitomo, K., Griesenbach, U., Inoue, M., Somerton, L., Meng, C., Akiba, E., Tabata, T., Ueda, Y., Frankel, G.M., Farley, R., et al. (2010). Toward gene therapy for cystic fibrosis using a lentivirus pseudotyped with Sendai virus envelopes. Mol. Ther. *18*, 1173–1182.
14. Munday, R.J., Coradin, T., Nimmo, R., Lad, Y., Hyde, S.C., Mitrophanos, K., and Gill, D.R. (2022). Sendai F/HN pseudotyped lentiviral vector transduces human ciliated and non-ciliated airway cells using alpha 2,3 sialylated receptors. Mol. Ther. Methods Clin. Dev. *26*, 239–252.
15. Steward, W.P., Scarffe, J.H., Austin, R., Bonnem, E., Thatcher, N., Morgenstern, G., and Crowther, D. (1989). Recombinant human granulocyte macrophage colony stimulating factor (rhGM-CSF) given as daily short infusions–a phase I dose-toxicity study. Br. J. Cancer *59*, 142–145.
16. Lund-Palau, H., Juarez-Molina, C.I., Meng, C., Bhargava, A., Pilou, A., Aziz, K., Clarke, N., Atsumi, N., Ashek, A., Wilson, M.R., et al. (2022). Correction of a chronic pulmonary disease through lentiviral vector-mediated protein expression. Mol. Ther. Methods Clin. Dev. *25*, 382–391.
17. Southam, D.S., Dolovich, M., O'Byrne, P.M., and Inman, M.D. (2002). Distribution of intranasal instillations in mice: effects of volume, time, body position, and anesthesia. Am. J. Physiol. Lung Cell. Mol. Physiol. *282*, L833–L839.
18. Donnelley, M., Morgan, K.S., Siu, K.K.W., and Parsons, D.W. (2013). Variability of in vivo fluid dose distribution in mouse airways is visualized by high-speed synchrotron X-ray imaging. J. Aerosol Med. Pulm. Drug Deliv. *26*, 307–316.
19. Djupesland, P.G. (2013). Nasal drug delivery devices: characteristics and performance in a clinical perspective-a review. Drug Deliv. Transl. Res. *3*, 42–62.
20. Schüepp, K.G., Straub, D., Möller, A., and Wildhaber, J.H. (2004). Deposition of aerosols in infants and children. J. Aerosol Med. *17*, 153–156.
21. Becquemin, M.H., Swift, D.L., Bouchikhi, A., Roy, M., and Teillac, A. (1991). Particle deposition and resistance in the noses of adults and children. Eur. Respir. J. *4*, 694–702.
22. Archiza, B., Leahy, M.G., Kipp, S., and Sheel, A.W. (2021). An integrative approach to the pulmonary physiology of exercise: when does biological sex matter? Eur. J. Appl. Physiol. *121*, 2377–2391.
23. Dominelli, P.B., Ripoll, J.G., Cross, T.J., Baker, S.E., Wiggins, C.C., Welch, B.T., and Joyner, M.J. (2018). Sex differences in large conducting airway anatomy. J. Appl. Physiol. *125*, 960–965.
24. Demicheli, R., Jirillo, A., Bonciarelli, G., Lonardi, F., Pradella, M., and Balli, M. (1989). Factors causing dose variability in drug administration. Cancer Chemother. Pharmacol. *24*, 363–366.
25. Kravitz, R.L., Duan, N., and Braslow, J. (2004). Evidence-based medicine, heterogeneity of treatment effects, and the trouble with averages. Milbank Q. *82*, 661–687.
26. Qi, T., and Cao, Y. (2023). Dissecting sources of variability in patient response to targeted therapy: anti-HER2 therapies as a case study. Eur. J. Pharm. Sci. *186*, 106467.
27. Saha, S., Doe, C., Mistry, V., Siddiqui, S., Parker, D., Sleeman, M., Cohen, E.S., and Brightling, C.E. (2009). Granulocyte-macrophage colony-stimulating factor expression in induced sputum and bronchial mucosa in asthma and COPD. Thorax *64*, 671–676.
28. Chen, W., Feng, X., Yao, L.K., Li, X., Yang, Z.M., Qin, X.Y., Li, Y., and Qiu, Y. (2025). Exogenous GM-CSF therapy for autoimmune pulmonary alveolar proteinosis: a systematic literature review. Front. Med. *12*, 1552566.
29. Uchida, K., Nakata, K., Suzuki, T., Luisetti, M., Watanabe, M., Koch, D.E., Stevens, C.A., Beck, D.C., Denson, L.A., Carey, B.C., et al. (2009). Granulocyte/macrophage-colony-stimulating factor autoantibodies and myeloid cell immune functions in healthy subjects. Blood *113*, 2547–2556.
30. Morens, D.M., Taubenberger, J.K., and Fauci, A.S. (2023). Rethinking next-generation vaccines for coronaviruses, influenzaviruses, and other respiratory viruses. Cell Host Microbe *31*, 146–157.
31. Savelkoul, H.F.J., Ferro, V.A., Strioga, M.M., and Schijns, V.E.J.C. (2015). Choice and Design of Adjuvants for Parenteral and Mucosal Vaccines. Vaccines (Basel) *3*, 148–171.
32. Sánchez Montalvo, A., Gohy, S., Rombaux, P., Pilette, C., and Hox, V. (2022). The Role of IgA in Chronic Upper Airway Disease: Friend or Foe? Front. Allergy *3*, 852546.
33. Harkema, J.R., Carey, S.A., and Wagner, J.G. (2006). The nose revisited: a brief review of the comparative structure, function, and toxicologic pathology of the nasal epithelium. Toxicol. Pathol. *34*, 252–269.

34. Griesenbach, U., McLachlan, G., Sinadinos, A., Cheminay, C., Ashour, J., Cox, J., Schwotzer, D., Vyhnal, K., Gupta, A., Chan, M., et al. (2026). F/HN-pseudotyped lentiviral vector efficiently transduces non-human primate airways with no evidence of relevant toxicity. Mol. Ther. Adv. *34*, 201655.

35. Carter, P.J., and Rajpal, A. (2022). Designing antibodies as therapeutics. Cell *185*, 2789–2805.

36. Limberis, M.P., Adam, V.S., Wong, G., Gren, J., Kobasa, D., Ross, T.M., Kobinger, G.P., Tretiakova, A., and Wilson, J.M. (2013). Intranasal antibody gene transfer in mice and ferrets elicits broad protection against pandemic influenza. Sci. Transl. Med. *5*, 187ra72.

37. Adam, V.S., Crosariol, M., Kumar, S., Ge, M.Q., Czack, S.E., Roy, S., Haczku, A., Tretiakova, A., Wilson, J.M., and Limberis, M.P. (2014). Adeno-associated virus 9-mediated airway expression of antibody protects old and immunodeficient mice against influenza virus. Clin. Vaccine Immunol. *21*, 1528–1533.

38. Focosi, D., McConnell, S., Casadevall, A., Cappello, E., Valdiserra, G., and Tuccori, M. (2022). Monoclonal antibody therapies against SARS-CoV-2. Lancet Infect. Dis. *22*, e311–e326.

39. Hyde, S.C., Pringle, I.A., Abdullah, S., Lawton, A.E., Davies, L.A., Varathalingam, A., Nunez-Alonso, G., Green, A.M., Bazzani, R.P., Sumner-Jones, S.G., et al. (2008). CpG-free plasmids confer reduced inflammation and sustained pulmonary gene expression. Nat. Biotechnol. *26*, 549–551.

40. Mann, P.C., Vahle, J., Keenan, C.M., Baker, J.F., Bradley, A.E., Goodman, D.G., Harada, T., Herbert, R., Kaufmann, W., Kellner, R., et al. (2012). International harmonization of toxicologic pathology nomenclature: an overview and review of basic principles. Toxicol. Pathol. *40*, 7S–13S.

41. Schneider, C.A., Rasband, W.S., and Eliceiri, K.W. (2012). NIH Image to ImageJ: 25 years of image analysis. Nat. Methods *9*, 671–675.

OMTA, Volume *34*

Supplemental information

Using the nose as a factory

to secrete proteins

into the lungs or circulation

Anthony Sinadinos, Robyn Bell, Claudia Ivette Juarez-Molina, Cuixiang Meng, Emily Castells, Mariana A. Viegas, Deborah R. Gill, Stephen C. Hyde, Uta Griesenbach, and Eric W.F.W. Alton

Table S1. Cellular level pathology parsed from haematoxylin and eosin stained transverse nasal-tissue sections from multiple small volume lentiviral vector (F.H/N), saline (TSSM), and untreated (sham) individualised mice, scored at 1 day post dosing. Nasal tissue pathology summarised for each mouse spanning five transverse levels of nose. Key: 0 normal and clear; + very limited and focal immune infiltrate; ++ more pronounced but focal infiltrate; +++ multifocal and/or infrequent prominent infiltrate. '/' indicated borderline representation. x focal blebbing with occasional loss of cells and/or infrequent keratinisation; xx more pronounced but sporadic blebbing and cell loss and/or pronounced keratinisation; xxx multifocal and pronounced blebbing and cell loss. Grades: 0 = within normal limits; 1 = mild focal structural changes with some to no inflammation; 2 = moderate range of mucosal changes with mild focused inflammation.

Nasal tissue									
		Sample	Frontal Nares (structure)	Lower level mucosa (structure)	Sub-mucosal blood vessel dilation	Macrophages	Neutrophils	Lymphocytes	Grade
1 day	Anaesthesia only	sham_1	0	0	-	0	0	0	0
		sham_2	0	xx	-	0	++	0	1-2
		sham_3	0	0	-	0	++	0	2
		sham_4	0	xx	-	0	++	0	2
		sham_5	0	0	-	0	++	0	1-2
	10 x 5µL over 50mins	F.H/N_1	x (keratinisation)	0	yes	0	++	++	2
		F.H/N_2	0	0	-	0	++	++	2
		F.H/N_3	0	0	yes	0	0	0	0-1
		F.H/N_4	0	xx	yes	0	0	0	1-2
		F.H/N_5	0	0	-	0	0	0	0
		TSSM_1	0	0	-	+	++	++	1
		TSSM_2	0	0	yes	0	++	0	2
		TSSM_3	0	xx	-	0	++	++	2
		TSSM_4	0	0	-	0	+	0	1
		TSSM_5	0	0	-	0	0	0	0

Table S2. Cellular level pathology parsed from haematoxylin and eosin stained lung transverse tissue sections from multiple small volume lentiviral vector (F.H/N), saline (TSSM), and untreated (sham) individualised mice, scored at 1 and 7 days post dosing. Lung tissue pathology summarised for each mouse spanning six transverse levels. Key: 0 normal and clear; + very limited and focal immune infiltrate; ++ more pronounced but focal infiltrate; +++ multifocal and/or infrequent prominent infiltrate. '/' indicated borderline representation. x mild and infrequent wall or extracellular space thickening; xx more pronounced but sporadic thickening. Grades: 0 = within normal limits; 1 = limited mild severity/top end of normal changes; 2 = limited severity.

<table>
<tr><th colspan="11">Lungs</th></tr>
<tr><th colspan="2"></th><th>Sample</th><th>Trachea</th><th>Lung macrophages</th><th>Lung neutrophils</th><th>Lung lymphocytes</th><th>Eosinophilia</th><th>Alveoli</th><th>Bronchial lymph nodes</th><th>Grade</th></tr>
<tr><td rowspan="13">1 day</td><td rowspan="3">Anaesthesia only</td><td>sham_1</td><td>0</td><td>0</td><td>0</td><td>+</td><td>0</td><td>0</td><td>0</td><td>0-1</td></tr>
<tr><td>sham _2</td><td>0</td><td>+</td><td>0</td><td>+</td><td>0</td><td>0</td><td>0</td><td>0-1</td></tr>
<tr><td>sham_3</td><td>0</td><td>0</td><td>0</td><td>0</td><td>0</td><td>0</td><td>0</td><td>0</td></tr>
<tr><td rowspan="10">10 x 5µL over 50mins</td><td>F.H/N_1</td><td>0</td><td>0</td><td>0</td><td>+</td><td>0</td><td>0</td><td>0</td><td>1</td></tr>
<tr><td>F.H/N_2</td><td>0</td><td>++</td><td>0</td><td>+</td><td>+</td><td>x</td><td>0</td><td>1-2</td></tr>
<tr><td>F.H/N_3</td><td>0</td><td>+</td><td>0</td><td>+</td><td>0</td><td>0</td><td>0</td><td>1</td></tr>
<tr><td>F.H/N_4</td><td>0</td><td>+</td><td>0</td><td>0</td><td>0</td><td>0</td><td>0</td><td>1</td></tr>
<tr><td>F.H/N_5</td><td>0</td><td>+</td><td>0</td><td>0</td><td>0</td><td>x</td><td>0</td><td>1</td></tr>
<tr><td>TSSM_1</td><td>0</td><td>0</td><td>0</td><td>+</td><td>0</td><td>0</td><td>0</td><td>0-1</td></tr>
<tr><td>TSSM_2</td><td>0</td><td>0</td><td>0</td><td>+</td><td>0</td><td>0</td><td>0</td><td>0</td></tr>
<tr><td>TSSM_3</td><td>0</td><td>0</td><td>0</td><td>+</td><td>0</td><td>0</td><td>+ (w/ vacuolation)</td><td>0</td></tr>
<tr><td>TSSM_4</td><td>0</td><td>0</td><td>0</td><td>+</td><td>0</td><td>0</td><td>0</td><td>0</td></tr>
<tr><td>TSSM_5</td><td>0</td><td>0/+</td><td>0</td><td>+</td><td>0</td><td>0</td><td>0</td><td>0-1</td></tr>
<tr><td rowspan="8">7 days</td><td rowspan="5">Anaesthesia only</td><td>sham_1</td><td>0</td><td>0</td><td>0</td><td>0</td><td>0</td><td>0</td><td>Not scored</td><td>0</td></tr>
<tr><td>sham_2</td><td>+</td><td>0</td><td>0</td><td>+</td><td>0</td><td>0</td><td>0</td><td>0-1</td></tr>
<tr><td>sham_3</td><td>0</td><td>0</td><td>0</td><td>0</td><td>0</td><td>0</td><td>+</td><td>0</td></tr>
<tr><td>sham_4</td><td>0</td><td>0</td><td>0</td><td>0</td><td>0</td><td>0</td><td>+</td><td>0</td></tr>
<tr><td>sham_5</td><td>0</td><td>0</td><td>0</td><td>++</td><td>0</td><td>0</td><td>Not scored</td><td>1</td></tr>
<tr><td rowspan="3">10 x 5µL over 50mins</td><td>F.H/N_1</td><td>0</td><td>0</td><td>0</td><td>+</td><td>0</td><td>0</td><td>++</td><td>0-1</td></tr>
<tr><td>F.H/N_2</td><td>0</td><td>0</td><td>0</td><td>++</td><td>0</td><td>0</td><td>Not scored</td><td>1</td></tr>
<tr><td>F.H/N_3</td><td>0</td><td>0</td><td>0</td><td>0</td><td>0</td><td>0</td><td>Not scored</td><td>0</td></tr>
</table>

		F.H/N_4	0	0	0	0	0	0	+	0
		F.H/N_5	0	0	0	+	0	0	+	1

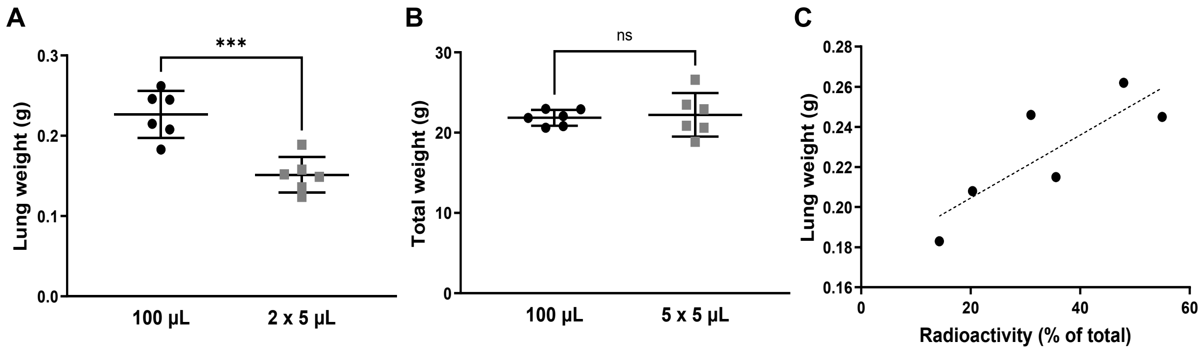

Figure S1. Small volume intranasal dosing of ^{99m}Tc-DTPA restricts fluid distribution to the site of instillation. Female 8-12 week-old mice received either a single 100μL, or two 5μL administrations (separated by 5 minutes) of volume(s) of PBS containing 5MBq of ^{99m}Tc-DTPA by intranasal bolus 'sniffing', and the separate body-areas, head, lungs, and the remaining body, were subsequently measured for radiotracer retention by gamma scintillation. Following dosing and culling, excised (A) lung (t-test, n=6, $p<0.001$) and (B) remaining total (head and body) (t-test, n=6, p=ns) weights were compared (individual values shown along with mean ± SD). (C) For tissue from the 100μL-dosed mice, lung weights were correlated with radioactivity (Pearson, r=0.832, r^2=0.6923, p=0.04). *** = $p<0.001$; ns = non-significant.

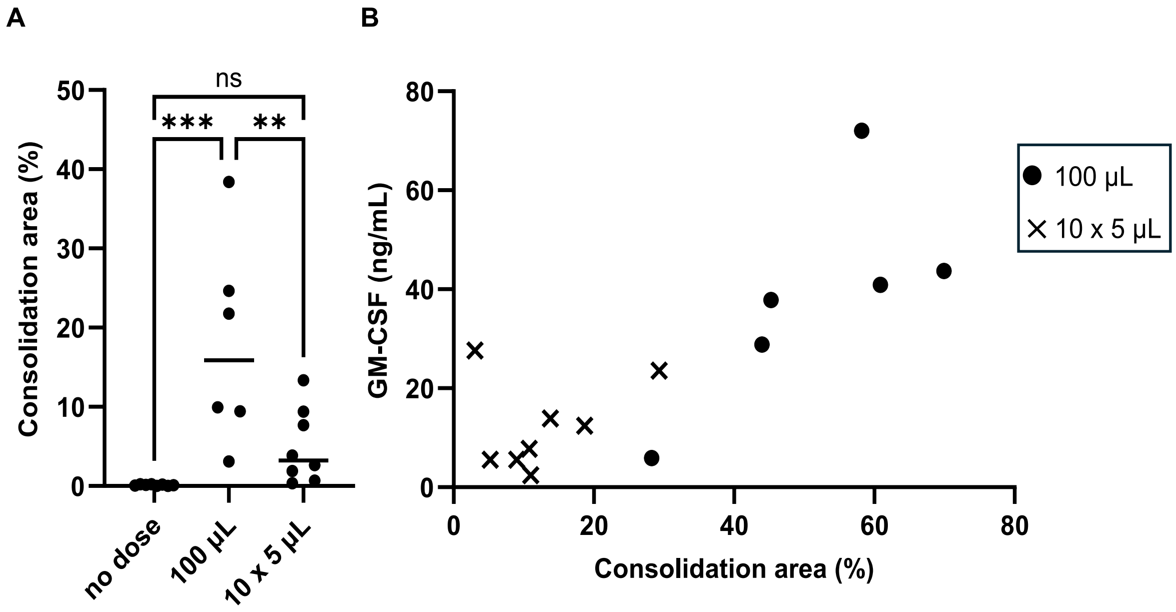

Figure S2. Granulocyte macrophage colony stimulating factor (GM-CSF) related lung consolidation, in Pulmonary Alveolar Proteinosis (PAP) mice, receiving 2.3e8 TU of rSIV.F/HN, encoding a hCEF-driven mouse codon-optimised version of GM-CSF, in either 100 µL or multiple small volume (10 x 5 µL) intranasal applications, measured 2.5 weeks post-dosing. (A) Whole tissue cross-sections from right lung lobes were analysed for consolidated areas of lung (ANOVA, n=8,6,8, F=12.2, DFn=2, DFd=19, p=0.0008: Tukey's post-hoc) (individual values shown along with mean). (B) Left and right lung average consolidation area was compared with bronchoalveolar lavage fluid GM-CSF levels measured from the same lentiviral vector treated mice (Pearson, n=14, r=0.8048, p=0.0005). ** = p<0.005, *** = p<0.0005, ns = non-significant.
